# Cost challenges for laboratory medicine automation in Africa

**Published:** 2010-09-18

**Authors:** Tanyanyiwa Donald Moshen

**Affiliations:** 1 Chris Hani Baragwanath Hospital NHLS Complex, Baragwanath Hospital NHLS Complex, Division of Chemical Path, South Africa

**Keywords:** Laboratory, Automation, analysers

## Abstract

**Abstract:**

Automation in laboratory medicine is inevitable and the only way forward especially in Africa where the staff turnover is high due to migration of experienced staff to Europe and America. Described here are the common issues that laboratory Managers and Directors encounter when upgrading, replacing analytical systems as well as daily running of diagnostic laboratories. The rapid advancement driven by the first world where research facilities, resources and expertise are available has seen changes in the both the hardware and software utilised by analyzers every two to three years. The downside is that in the process of replacing/phasing out old analysers, the first world countries in some cases donate them to second and third world countries as refurbished analysers. Problems in obtaining spares ensue since the production of new analysers results in reduced production or even of old spares. Unavailability or delayed availability of spares results in suspension of diagnostic service by the recipient laboratory. In some areas costly modifications to the analysers or the location/building have had to done to suite local (African) conditions, hence the need for Laboratory managers to understand fully the analysers’ operational requirements before purchasing or accepting donations.

## Background

Total automation in diagnostic medicine has resulted in rapid advancement in the hardware and software of clinical laboratory analyzers [1]. Almost instant diagnosis by a clinician or pathologist calls for quick, reportable and accurate results, utilizing cost effective means. Even point of care analysers have advanced from being single test to complex multiple test analysers. Therefore several models available offer fully automated clinical analyzers constituting three major components: 1) sample probe, an equivalent of robotic arms, 2) several different types of detectors that measure results of the reactions between reagents and samples and 3) a microprocessor that converts the electronic readings into reportable values. According to Frost & Sullivan Research Associate Arun AK [2] a lack of clarity about laboratory automation is hampering market expansion. Although laboratories are convinced that automating their processes is the only way forward, they remain relatively ignorant about the exact means by which to achieve this goal. “The growth of the European clinical lab automation market is mainly dependent on the stand-alone and total lab automation markets“ [2]. Increased cost pressures and rising testing volumes are being exacerbated by a shortage of trained technical personnel, automation solutions are coming to the fore due to their ability to reduce both labour and operating costs in clinical laboratories as well as improve patient care [3].

Total automation is the ultimate for Africa because of the increased urgent demand for diagnostics results in the face of mass exodus of experienced medical technologists in search for better standard of living offered by the northern hemisphere. However automation in Africa will inevitably cost twice if not thrice as much as in Europe not because of the import and shipping costs but mainly because the pace is dictated in Europe and by the time we are getting familiar with one analyzer, a new one with better features and more “user friendly” will be on promotion. Hence parts and consumables for the old one (3 years old) will start running low therefore we are left with no option but to abandon the analyzer in order to maintain a seamless service or risk running out of reagents for outdated (3 years old) machines.

The rapid change of analysers and the chronic problems faced made the lease/reagent use placement policy look attractive. In such an agreement, which is heavily supported by vendors, the responsibility rests with the vendor to maintain the analyser but the cost of reagents and consumables is running at costs more than the purchase of ten analysers by the time the (new) old analyser is phased out. The epidemical levels of HIV have seen a parallel increase in the number of tests in all the departments resulting in the need to automate in order: 1) to obtain accurate results for such huge numbers, 2) to protect the analysts/technologists from cross infection during analysis, 3) need to make urgent diagnosis with short turn-around times that could not be achieved with manual or semi automated methods. The global high turn-over of technologists with the need to maintain the high quality of results requires automation in order not to compromise the above.

## Problems faced by analysers in Africa

**Water**

Frequent pipe busts often compromise quality and supply of water, which results in the inlet water after resumption of supply not being of the analar grade but commercial grade. This has triple effects in that the water filter on the analyser is over-worked in order to maintain the grade required to run the analyser. This will results in filters requiring frequent changes and thus one filter change in Europe equals three filter changes in Africa. That alone triples the filter budget for a laboratory in Africa compared to Europe or in the rural area compared to city laboratories.

The second effect is that even though water for the reconstitution/ preparation of lyophilized reagents now passes through water purification systems that include deionisers and UV treatment, for some reason the water does not compare with the ‘boiling’ distillation followed by deionisation that used to be employed before. In most cases the reagents work well “so to speak” soon after filter changes with progressively more quality control and precision check failures being overcome by repeated recalibrations in order to achieve the accepted Westgard rules for accepting the results hence avoid litigation. So essentially besides the filters, that laboratory has used additional calibrators and control materials to release results. The solution would be water filters or machines destined for Africa to have double water cleaning systems. A tall order but it would go a long way to install miniature water recycling/purifying systems similar to those used by the water department. This is particularly important in that water is a very scarce commodity in some parts of the world especially in Africa.

In addition to the above two effects of water quality and interrupted supply, the importance and the need/urgency of an inbuilt water reservoir and purification technology is noted in that the analysers do not resume function soon after resumption of water supply because there is need for pressure to built up to the calibrated levels. Most importantly patient samples have to be exported to others laboratories during the repair period and this has a negative effect on turn-around time and hence optimal patient management is compromised as the exercise of transporting samples results in misplacement of sample, delay in analysis due to road traffic congestion. Therefore again unlike in the well resourced countries the analysers in Africa and more so in rural overuse the pressure controlling units and hence will need more frequent replacements and have additional transport costs.

**Electricity**

Most analysers have uninterruptible power backup systems/units that protect the electronic circuits during the transition from normal electricity supply to the generator power takes over during that surge. Even though this appears to be a safe way of maintaining continuity, there is no scientific study or record that investigated the effect of power surges on the electronic calibrations and settings of analysers. It is very evident that the vendor’s electronic engineers or application specialists are more frequently seen in those laboratories located in rural type environment. Some institutions do not have generators and so they resort to physically switching off the analysers in order to minimise the damage during the upsurge when the power comes back. There are also instances when electrical power has been reported to be unavailable for periods, too long to be sustained by generators. The solution would be for such institutions o to install solar power that is linked to invertors connected to the PBU so that the analyser does not experience any interruptions at all. Besides the effects on the analysers, the prolonged power cuts have a major effect on the stability of reagents that require refrigeration. The calibrators that are usually frozen will under-go thawing and freezing without realization. Unknown to the user the reagent would still be viable because of the valid expiry date but the analyser will not calibrate resulting in further calibrations and even purchasing of a new lot of calibrators. Closer to home there are laboratories that have lost substantial amounts of money due to prolonged power cuts rendering cartridges on board some with 500 tests useless upon power supply resumption.

**Dust and Vibrations**

Africa is undergoing extensive constructions in terms of ordinary buildings and several earth, excavations for new structures are taking place including widening of roads. This results in a lot of dust and this inevitably ends up inside buildings and machines. The accumulation of dust despite regular cleaning will reduce both the efficiency of the analyser and its life span.

The dust collection was tested between Johannesburg General and Chris Hani General Hospitals. The commonest problem is the blockage of the micro tubes even though they are supposed to be sealed. The plastic dust covers that are supplied together with the analysers do not seem to be effective in preventing dust from settling on the analysers. The dynamite induced vibrations do not seem to do any major harm, and without rector scale measurements it would be difficult to comment except to say that laboratories above the underground train system route noted frequent both chemical and electronic calibrations especially during the blasting week. This seemed to suggest that such minor vibrations cause some form of disruptions for unknown/unexplainable reasons.

**Personnel**

The rural laboratories or Africa are the worst affected due to migration of experienced technologists move to more lucrative environments with better working conditions [4]. However this problem is not unique to Africa as literature review revealed that shortage of medical technologists is a worldwide problem, which has also seen the formulation of the Medical Laboratory Personnel Shortage Act HR 623 [5]. The US Department of Labour projects that 13,200 medical laboratory professionals will be needed yearly through 2010 [5]; unfortunately, fewer than 5,000 graduate from accredited training programs each year [5] Another concern is the average age of the laboratory workforce, which has been rising steadily [5]. However in South Africa the situation is different in that a lot of the younger laboratorians are using the training as a stepping-stone to enter into other health related fields or go overseas. Well resourced institutions are like Beth Israel Deaconess Medical Center, Children’s Hospital Boston and New England Baptist Hospital are partnering with Bunker Hill Community College and the Boston Private Industry Council on an initiative to train current hospital employees to be Medical Laboratory Technicians. In an employer-designed and led response to a critical need to staff this profession, the hospitals are working with Bunker Hill Community College to build and accredit a Medical Laboratory Technician program in the greater Boston area, which currently has no such training program [6].

Other institutions like CH Bara are faced with the unique problem of technologists finding it as an excellent training centre but immediately leave at the end of the contractual agreement to join smaller laboratories within the same organisation (less workload at the same salary). So the human resource element is the major driving force making automation inevitable, more so in Africa. The experienced technologists who can easily diagnose the problems and commence troubleshooting are leaving, creating a situation where inexperienced staff and students are left to provide service. However this often results in more damage during their determined efforts of trial and error in trying to make the diagnosis. The Users Manuals often refers one to another section in order to solve an encountered problem and that causes a lot of distress on the part of the inexperienced individual, hence the call for more user-friendly step-by-step approach for every possible problem that can be encountered.

**Affiliated Laboratories**

The acquisition or the use of similar analysers from the same vendor makes economic sense and is also considered to have the advantage in that all members of staff will understand the analysers better especially with agreements that may see an exchange or mere relief of members of staff members between laboratories. The inter-transfer of patients between hospitals for specialised treatment requires the movement of the folders with pathology results and hence the need for standardisation/uniformity of analysers in order to maintain similar interpretation by different health workers who may attend to the same patient for different consultations. However the advent of uncertainty of measurements invalidates the need to have similar analysers by sister laboratories. The other disadvantage is that if a particular element or reagent is of sub standard or out of production as happened with a reagent at another laboratory, it all the tests will be sent away. The elements of after sales efficiency due to competition are eradicated once a single vendor supplies the whole company with its analysers. Efficiency is maintained if the vendor is aware that they can be replaced any time.

**Tender Procedures**

In most cases, vendors would like an opportunity to highlight on paper the special features of their products. However most of them are reluctant to provide clear and categorical answers related to service calls, the cost of replacement parts, the number of instruments currently in service and the specific location. Issues related to guarantee of prices for reagents and other consumables and to estimate the reasonable yearly usage costs associated with operating each instrument. Its becoming common practice in Europe for vendors to leave their analyzers in the laboratory for a few days so that your staff can assess the instrument at their convenience in the absence of an eager representative and then a comparison charts are drawn up to reflect at a glance the comparative benefits of every instrument being considered. This may sound impossible or impractical but if life ending automobiles are freely given out for test drives, surely life saving equipment deserves theoretical tender presentation followed by 1-2 months (test drive) practical presentation/placement which will include evaluation at the vendor’s expense. The practical presentation will effectively assess the downtime periods and the availability of after sales service, which is often not possible to evaluate in a theoretical presentation. The time frame between the automobile test drive and the analyser is to scale from starting, running, braking and stopping.

## Conclusion

We have successfully completed what is probably the largest Auto Laboratory in Southern Africa. Despite the mechanical aspect being perfect there were several delays due to incomplete validations which had to be repeated due to problems encountered with water and electrical supply. Our validation budget was more than double what had been focused. During the budget preparation all the above factors cannot be translated into figures and therefore cannot be included in the budget. However evidence is only seen if one compares and contrasts two institutions with similar analysers but located in two different worlds so to speak. In most cases the one located in an African type of environment will register a deficit or lower profit margin than the other.

**What Is an Ideal Analyser?**

From the above discussion it is clear that state of art analyser is area and user dependent and hence will not be ideal for all the laboratories. Therefore an ideal analyser would be one that: 1) Calibrates as scheduled, 2) minimal accurate and precision repeat check runs at scheduled times, 3) Results output as per analytical laid turnaround times, 4) Minimal down times, 5) 80% capacity being adequate to meet demands of the institute being served with the 20% being reserved for prospective increased load with no effect on turnaround time, 6) finally the Unit Management Committee best defines the ideal analyser. Despite the high tech (state of the art) automation in the first world (Europe and Americas) legislature has been put in place to train more medical technologists. It therefore becomes only a myth that total automation will solve/replace the human resource requirements in the under resourced parts of the world. Secondly the above discussion doubts the merits of harmonising services/analysers if competition is to be encouraged.

## Acknowledgements

I wish to thank Dr. Nontombi Mbelle, the National Health Laboratory Services, and Central Region Executive Manager for encouraging me to analyse the causes of the down times for our analyzers and problems we encountered during the evaluation exercises for the new tests and analyzers introduced in our department.
